# Utility of periodic medical questionnaires and examinations for immune-related adverse event screening: A prospective observational study

**DOI:** 10.1371/journal.pone.0274451

**Published:** 2022-09-29

**Authors:** Takeshi Azuma, Masato Kano, Shohei Iwata, Sachi Honda, Yuji Miyoshi, Junko Nishiguchi

**Affiliations:** 1 Division of Urology, Tokyo Metropolitan Tama Medical Center, Fuchu, Tokyo, Japan; 2 Department of Rheumatic Diseases, Tokyo Metropolitan Tama Medical Center, Fuchu, Tokyo, Japan; 3 Division of Nursing, Tokyo Metropolitan Tama Medical Center, Fuchu, Tokyo, Japan; IRCCS Giovanni Paolo II Cancer Hospital, ITALY

## Abstract

**Background:**

Immune checkpoint inhibitors (ICIs) are associated with immune-related adverse events (irAEs) specific to the immunity-boosting activity of the drugs and may necessitate discontinuation of treatment depending on their severity. IrAEs may be difficult to diagnose in their early stages as they can occur in any organ. The present, prospective, observational study is the first to attempt to assess the utility of periodic medical questionnaires and laboratory, radiological, and physiological examinations in diagnosing irAEs.

**Methods:**

We analyzed 51 patients who received immunotherapy for metastatic renal or urothelial carcinoma at Tokyo Metropolitan Tama Medical Center between 2016 and 2020. A medical questionnaire consisting of 41 questions and laboratory tests were administered to the patients on the day of each ICI administration and 1 week afterwards. A significant complaint was defined as a complaint not addressed in the questionnaire immediately prior to the first ICI administration.

**Results:**

Fifty-one patients with metastatic renal or urothelial carcinoma were enrolled. The mean age was 72.1 years (range: 54–88 years). The male: female ratio was 32: 19. Of the total cohort, 26 (51%) patients had renal carcinoma, and 25 (49%) had urothelial carcinoma. The median follow-up time was 2.6 (range: 0.4–40.7) months. Thirty-three patients (65%) experienced irAEs.

**Conclusions:**

In our cohort, periodic medical questionnaires and examinations were effective for early diagnosis and prompt treatment of irAEs. Although periodic examinations led to a high irAE diagnosis rate, the attendant medical cost was high. Further study is needed to find ways of addressing this issue.

## Introduction

Immune checkpoint inhibitors (ICIs) are effective in treating several types of cancer [1–13]. However, despite their efficacy, they are associated with immune-related adverse events (irAEs), which are specific to the immunity-booting effects of ICIs. Depending on their severity, irAEs may require discontinuation of ICI therapy [14].

IrAEs may have dermatological [15], musculoskeletal [16], endocrinological [17], gastrointestinal [18], renal [19], cardiac [20] or pulmonary [21] manifestations and may be difficult to diagnose in their early stages as they can occur in any organ. Most patients are asymptomatic or have indefinite complaints while others may have carcinoma-like symptoms. Numerous cases of severe irAE and related fatalities have previously been reported. In these reports, the protocol used to evaluate the irAEs was unclear, but the severity of the patients’ symptoms prompted exhaustive investigation. Periodic medical questionnaires and examinations can provide a reliable method of mitigating irAEs by enabling prompt diagnosis and uniform treatment. They may also have the added benefit of providing accurate information about irAE incidence.

The present, prospective, observational study is the first to attempt to assess the utility of periodic medical questionnaires and laboratory, radiological, and physiological examinations in diagnosing irAEs.

## Materials and methods

### Patients

The present, prospective, observational study was conducted at Tokyo Metropolitan Tama Medical Center between 2016 and 2020. Fifty-one patients receiving immunotherapy for metastatic renal or urothelial carcinoma were enrolled. This study was approved by the ethical review board of Tokyo Metropolitan Tama Medical Center (30–135) and was conducted in accordance with the principles of the Declaration of Helsinki and the Good Clinical Practice Guidelines. Informed consent was obtained from all the participants.

### Medical questionnaire

**Fig 1:**
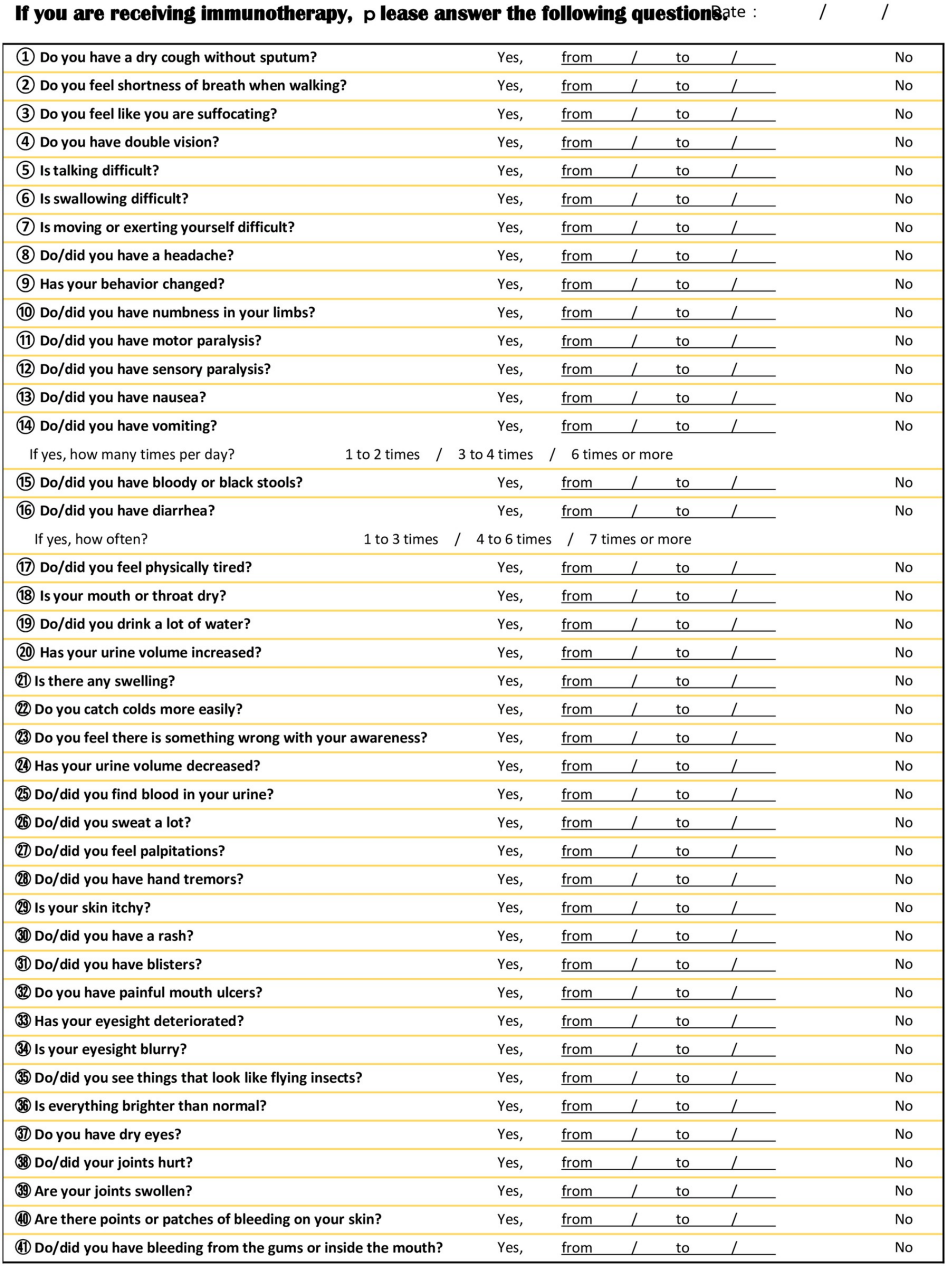
Medical questionnaire.

**Fig 2:**
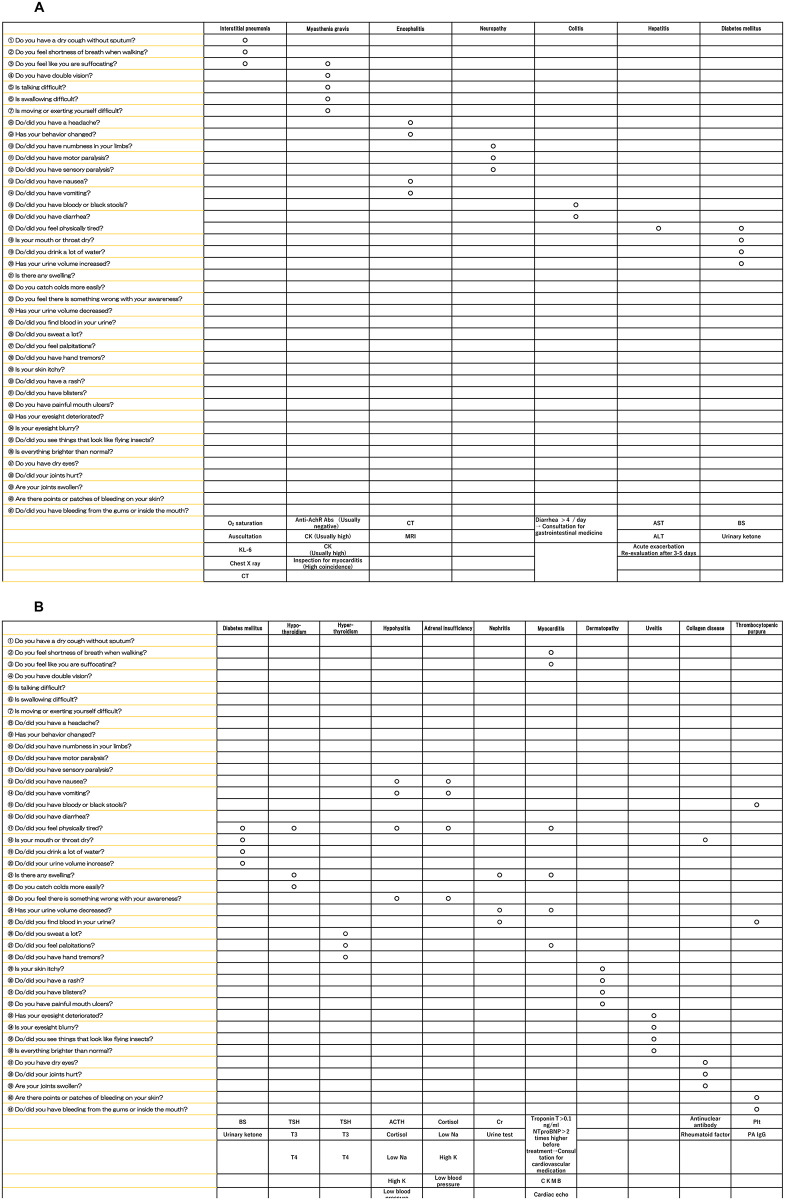
Reference chart corresponding to each question.

Figs 1 and 2 show the medical questionnaire consisting of 41 questions and a reference chart corresponding to each question, respectively. These questions were formulated by referring to reports of irAEs in the Checkmate 025 clinical trial [4]. The questionnaire was administered before the first ICI administration, on the day an ICI was administered, and 1 week later. The pre-ICI responses were used as the baseline against which the responses to the other 2 questionnaires were compared. A significant complaint was defined as a complaint not reported on the first questionnaire. Analysis of the irAEs was facilitated by using a reference chart of irAEs based on the items on the questionnaire (Fig 2).

### Examinations

As with the questionnaire, physiological and laboratory examinations were performed everyday an ICI was administered and 1 week later. Table 1 shows the examination details. A chest Xray and echocardiography was performed monthly and every 3 months, respectively.

**Table 1:** Examination details.

**Before immunotherapy**
CBC・Blood picture・AST・ALT・T-Bil・LDH・γGTP・TP・Alb
UN・Cre・UA・Na・K・Cl・Ca・CK
TSH・FT3・FT4・ACTH・Cortisol
Anti-thyroglobulin Abs・Anti-TPO Abs
KL-6・SP-D
BS・HbA1C
ANA・IgG・IgA・IgM・IgE
Anti-Ach-R Abs
HBs・HBc・HCV
PT・APTT・D-dimer NT-proBNP
Urine test
Two preserved serum samples
**During immunotherapy (every month)**
CBC・Blood picture・AST・ALT・T-Bil・LDH・γGTP・TP・Alb
UN・Cre・UA・Na・K・Cl・Ca・CK
TSH・FT3・FT4・ACTH・Cortisol
KL-6 NT-proBNP
BS
Urine test

## Results

### Patient characteristics

Table 2 summarizes the characteristics of the patients receiving ICI therapy. Fifty-one patients with metastatic renal or urothelial carcinoma were enrolled. Their mean age was 72.1 years (range: 54–88 years). The male-to-female ratio was 32: 19. Of the total, 26 (51%) patients had renal carcinoma, and 25 (49%) had urothelial carcinoma (bladder carcinoma: 13; upper urinary carcinoma: 12). The median follow-up time was 2.6 (range: 0.4–40.7) months.

**Table 2:** Patient characteristics.

		Renal Cell Carcinoma		Urothelial Carcinoma	All Patients
		Nivolumab (20)	Nivolumab	Pembrolizumab (25)	(51)
			+Ipilimumab (6)		
**Age**	**Average**	**72.8**	**72.4**	**71.8**	**72.1**
	**Range**	**56–88**	**56–83**	**54–83**	**54–88**
**Sex**	**Male**	**11 (55%)**	**1 (17%)**	**19 (76%)**	**31 (61%)**
	**Female**	**9 (45%)**	**5 (83%)**	**6 (24%)**	**20 (39%)**
**ECOG PS**	**0**	**19 (95%)**	**6 (100%)**	**25 (100%)**	**50 (98%)**
	**1**	**1 (5%)**	**0 (0%)**	**0 (0%)**	**1 (2%)**
**Clinical Response**	**CR**	**2 (10%)**	**0 (0%)**	**4 (16%)**	**6 (12%)**
	**PR**	**2 (10%)**	**2 (33%)**	**5 (20%)**	**9 (18%)**
	**SD**	**7 (35%)**	**2 (33%)**	**4 (16%)**	**13 (25%)**
	**PD**	**9 (45%)**	**2 (33%)**	**11 (48%)**	**23 (45%)**

ECOG PS: Eastern Cooperative Oncology Group Performance Status.

### Immune-related adverse events

Thirty-three patients (65%) experienced an irAE (Table 3). Table 4 shows the details of the irAEs. Three and eleven patients experienced 3 and 2 irAEs, respectively. In total, 50 irAEs were observed; in 2 patients they were Grade 1, in 20 patients they were Grade 2, and in 1 patient they were Grade 3. Whenever an abnormality was detected via periodic administration of the questionnaire and targeted examinations, more detailed testing was conducted. As a result, almost all irAEs were able to be detected before exceeding Grade 2 severity. There was only 1 case of colitis with Grade 3 diarrhea. Thus, the periodic administration of the medical questionnaire and targeted examinations were useful for detecting irAEs at an early stage and allowing prompt treatment.

**Table 3:** Immune-related adverse events.

Immune-related adverse event	Number of patients
Interstitial pneumonitis	5
Colitis	6
Hepatitis	10
Cholangitis	1
Type 1 diabetes mellitus	1
Thyroid dysfunction	2
Isolated ACTH deficiency	3
Dermatitis	12
Arthritis	1
Eosinophilia	7

**Table 4:** Details of immune-related adverse events.

	Renal Cell Carcinoma		Urothelial Carcinoma
	Nivolumab	Nivolumab+Ipilimumab	Pembrolizumab
**Interstitial Pneumonitis**			
**Grade 2**	3		2
**Colitis**			
**Grade 2**	1		4
**Grade 3**	1		
**Hepatitis**			
**Grade 1**	4	2	2
**Grade 2**			2
**Cholangitis**			
**Grade 2**			1
**Type 1 Diabetes Mellitus**			
**Grade 2**		1	
**Thyroid Dysfunction**			
**Grade 2**			1
**Isolated ACTH Deficiency**			
**Grade 2**	2		1
**Dermatitis**			
**Grade 1**	5	3	5
**Arthritis**			
**Grade 2**		1	
**Eosinophilia**			
**Grade 1**	1	2	4
**Grade 2**	1		
**Cardiomyopathy**			
**Grade 1**	1		

### Medical questionnaire

**Fig 3:**
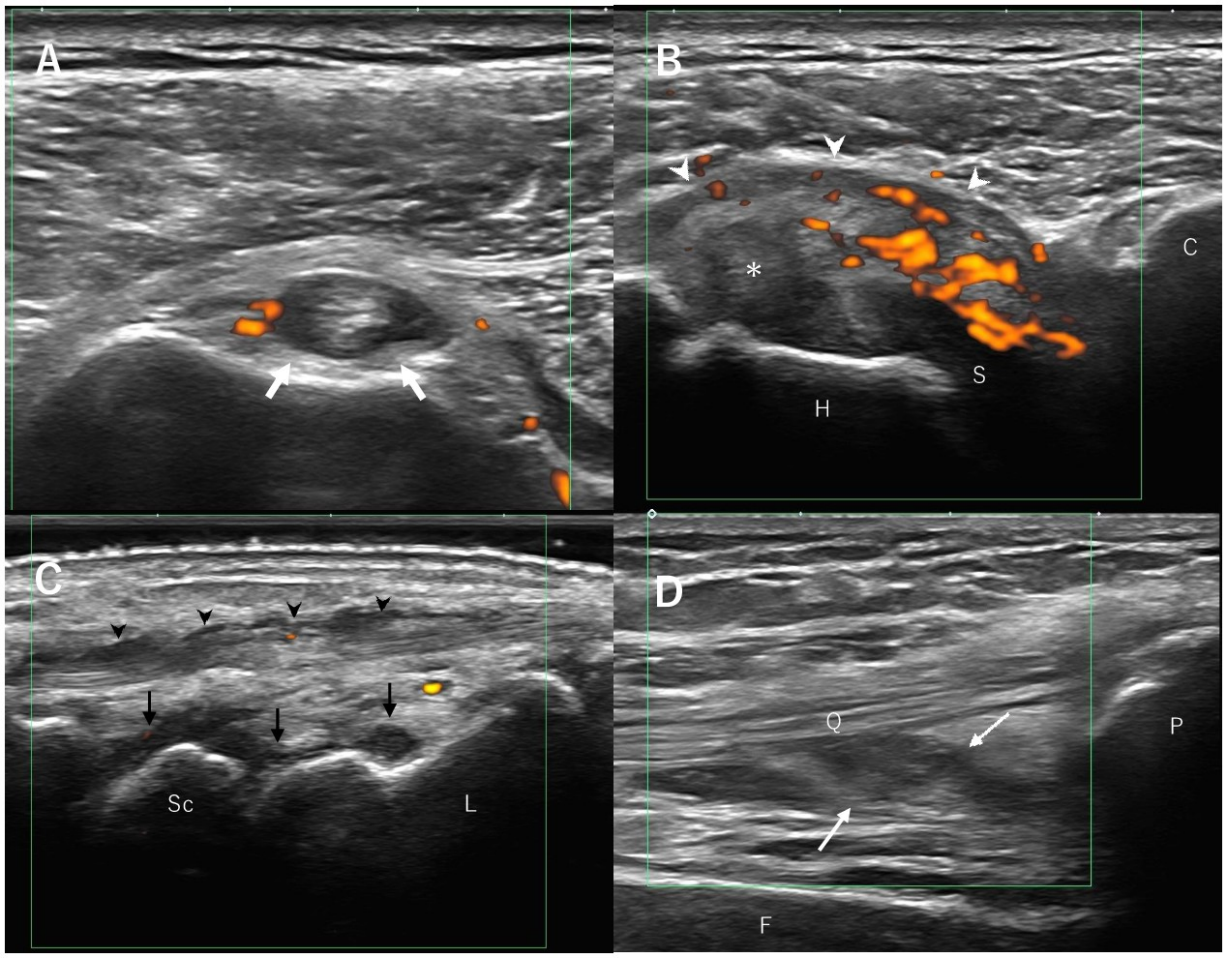
Musculoskeletal ultrasound images of the patient with polyarthritis induced by immuno-checkpoint inhibitors. A. Transverse imaging over the bicipital groove of the right humerus shows tenosynovitis of the long head of the biceps brachii (arrows) with moderate effusion and synovial hypertrophy within the tendon sheath. B. Transverse imaging over the right humeral lesser tuberosity shows prominent power Doppler signals extending from beneath the coracoid process over the subscapularis. The subdeltoid bursitis (arrowheads) and the swelling of the long head of the biceps brachii within the rotator interval (asterisk) can be seen. C. Sagittal imaging over the right dorsal wrist shows synovial hypertrophy at the dorsal recesses of the radiocarpal and midcarpal joints (black arrows). Tendinitis surrounding the extensor tendon can be seen (arrows). D. Sagittal imaging of the anterior knee shows hypoechoic to isoechoic synovial hypertrophy within the suprapatellar recess (arrows). H, humeral head; S, subscapularis; C, coracoid process; Sc. Scaphoid; L, lunate; F, femur; P, patella; Q, quadriceps tendon.

The medical questionnaire was administered 1008 times. Table 5 summarizes complaints following treatment with ICIs. No difference in the distribution of the complaints was found among patients receiving nivolumab, pembrolizumab or ipilimumab. The most frequent complaint was fatigue (14 / 51 or 27%). However, this complaint was not an irAE but a symptom of the cancer. Diarrhea, arthralgia, and itching raised the index of suspicion for colitis, arthritis, and dermatitis, respectively. In 6 patients with diarrhea, all underwent computed tomography and colonoscopy, received the diagnosis of colitis, and were started on oral prednisolone. Arthralgia developed in 1 patient. Although a serological examination returned negative for anti-CCP antibody and rheumatoid factor, joint ultrasonography detected joint effusion, synovial thickness, and power doppler signal (Fig 3). Based on these findings, the patient received a diagnosis of arthritis and started infliximab, methotrexate, and oral prednisolone therapy. Thirteen patients with pruritis received a diagnosis of dermatitis and started steroid ointment therapy. As can be seen, periodic medical questionnaires can play an important role in diagnosing colitis, arthritis, and dermatitis.

**Table 5:** Association between complaints and immune-related adverse events.

Systems	Number of Patients with Complaints	Number of Patients with irAE Diagnosis Based on Complaints
Respiratory	2	0
Neurological	8	0
Digestive	7	7
Endocrine	14	0
Cutaneous	12	12
Ocular	3	0
Musculoskeletal	3	1
Hematological	1	0

### Laboratory examination

Laboratory examinations led to the diagnosis of autoimmune cholangitis, hypothyroidism diabetes mellitus type-1, and isolated adrenocorticotropic hormone (ACTH) deficiency. The patients in whom irAE was diagnosed based on the laboratory examination findings did not experience any of the symptoms associated with the irAEs listed on the medical questionnaire. Prompt follow-up testing on the occurrence of an abnormal finding enabled early diagnosis and timely treatment before symptom development in all the affected individuals.

### Radiological examination

Radiological examination revealed interstitial pneumonitis in 5 patients who did not report any dyspnea-related symptoms on the questionnaire. Oximetry also failed to detect hypoxemia. Based only on the radiological findings, all these patients were successfully treated with oral corticosteroid (1 mg/kg/day), which was tapered as the pulmonary lesions improved.

### Physiological examination

In 1 patient, periodic echocardiography was able to detect a gradual ejection fraction which decreased by 30% over 3 months. Further investigation revealed cardiomyopathy. The patient was successfully treated with oral corticosteroid (1 mg/kg/day), which was tapered as the symptoms improved.

## Discussion

Periodic screening was able to detect some cases of irAE while the questionnaire and targeted examinations provided clues to detecting other types of irAE. Thanks to the periodic administration of the questionnaire and the examinations, the irAEs were able to be detected at an early stage. Routine laboratory, radiological, and physiological examinations were particularly useful in detecting irAEs in asymptomatic patients. These results indicated that periodic examinations have the potential to detect irAEs before they reach the severe stage. In fact, the actual frequency of irAEs was higher in our cohort than in previous reports.

Periodic administration of questionnaires and examinations offers various advantages. However, each is useful for detecting different types of irAE. The questionnaire was effective in detecting irAEs with characteristics unlike those of sporadic diseases. Only the medical questionnaire was useful for detecting irAEs of this type because these patients never have laboratory examinations findings typical of sporadic diseases. On the other hand, the periodic examinations were effective in detecting irAEs similar to the symptoms of sporadic diseases, thus enabling their treatment before symptom onset.

In the present cohort, the questionnaire was able to diagnose inflammatory arthritis in 1 female patient. Her serological examination was negative for rheumatoid factor (RF) and anticyclic citrullinated peptide (CCP) antibody, and she had the same titer of antinuclear antibodies (ANA) as before immunotherapy. Cappelli et al. reported 13 patients with inflammatory arthritis caused by immunotherapy [22], all of whom were negative for RF and CCP. ANA was positive in 3 patients, of whom only 1 had a high titer. Changes in ANA before and after immunotherapy were unknown in these 3 patients. Their report suggested that auto-antibodies were not useful for diagnosing inflammatory arthritis. On the other hand, imaging studies, such as ultrasonography or magnetic resonance imaging, are useful for diagnosing arthritis.

Another advantage of the periodic questionnaire was its educational value both for the patients and medical staff. Some of the patients called to report complaints that they had previously seen on the questionnaire, such as diarrhea, joint pain, and skin rash, which can provide some insurance against inexperienced residents or nurses omitting to ask about such complaints in an interview. Educating the patients and the medical staff may thus lead to earlier detection and treatment of irAEs.

Periodic examinations were also very effective in detecting irAEs at an early stage. In our cohort, all irAEs detected in a periodic examination were in their early stages before symptom onset. In the present study, ACTH deficiency was diagnosed in a patient on the basis of the findings of a periodic examination. The diagnosis of isolated ACTH deficiency is usually challenging; as a result, the disease develops insidiously until it causes hypoadrenalism, which in turn can lead to hypoglycemia, hypotension or hyponatremia and become fatal without treatment [23]. The incidence of irAEs associated with the pituitary gland was higher in patients receiving anti-CTLA-4 antibodies than anti-PD-1 antibodies. Several retrospective studies reported a low incidence of pituitary-related irAEs, which had a frequency of 0.5–1.6% and 2.7–5.2% after anti-PD-1 antibody and anti-CTLA-4 antibody therapy, respectively [24,25]. The present, prospective study found the frequency of pituitary-related irAEs to be 6.7% and 0% after monotherapy with anti-PD-1 antibody and anti-CTLA-4 antibody, respectively. The incidence was similar to that reported in a previous, prospective study (9.1%) [21]. Periodic endocrine evaluations were performed as in the previous study although they differed in 2, salient respects, which may have the potential to provide new information. First, endocrine tests were performed more frequently (monthly) in the present study, thereby allowing earlier detection of pituitary-related irAEs. Second, the questionnaire was used to ensure that symptoms, including irAEs, were not overlooked and to determine whether a given symptom was present at the onset of the pituitary-related irAEs.

Periodic examinations have the disadvantage of being costly to perform. Frequent examinations can unnecessarily increase the financial burden on the patients and healthcare system. Although they tend to improve the diagnosis rate by detecting asymptomatic irAEs, the latter may not require treatment. Treatments prescribed on the basis of such findings also may contribute to increasing medical costs unnecessarily.

The present study has a limitation. The study included a widely varied patient population from a single japanese institution and the total number of patients analyzed was relatively small.

## Conclusion

In the present cohort, periodic administration of a questionnaire and target examinations provided various advantages in detecting irAEs. Both were useful for early diagnosis and prompt treatment. However, the high diagnosis rate inflated medical costs. Further research is necessary to find the optimal balance of diagnosis and treatment-related costs.

## Supporting information

S1 Data (XLSX)

## References

[1] Borghaei H, Paz-Ares L, Horn L, Spigel DR, Steins M, Ready NE, et al. Nivolumab versus Docetaxel in Advanced Nonsquamous Non-Small-Cell Lung Cancer. N Engl J Med. 2015;373(17):1627–39. doi: 10.1056/NEJMoa1507643 26412456 PMC5705936

[2] Brahmer J, Reckamp KL, Baas P, Crino L, Eberhardt WE, Poddubskaya E, et al. Nivolumab versus Docetaxel in Advanced Squamous-Cell Non-Small-Cell Lung Cancer. N Engl J Med. 2015;373(2):123–35. doi: 10.1056/NEJMoa1504627 26028407 PMC4681400

[3] Weber JS, D’Angelo SP, Minor D, Hodi FS, Gutzmer R, Neyns B, et al. Nivolumab versus chemotherapy in patients with advanced melanoma who progressed after anti-CTLA-4 treatment (CheckMate 037): a randomised, controlled, open-label, phase 3 trial. Lancet Oncol. 2015;16(4):375–84. doi: 10.1016/S1470-2045(15)70076-8 . 25795410

[4] Motzer RJ, Escudier B, McDermott DF, George S, Hammers HJ, Srinivas S, et al. Nivolumab versus Everolimus in Advanced Renal-Cell Carcinoma. N Engl J Med. 2015;373(19):1803–13. doi: 10.1056/NEJMoa1510665 26406148 PMC5719487

[5] Motzer RJ, Tannir NM, McDermott DF, Aren Frontera O, Melichar B, Choueiri TK, et al. Nivolumab plus Ipilimumab versus Sunitinib in Advanced Renal-Cell Carcinoma. N Engl J Med. 2018;378(14):1277–90. doi: 10.1056/NEJMoa1712126 29562145 PMC5972549

[6] Sharma P, Retz M, Siefker-Radtke A, Baron A, Necchi A, Bedke J, et al. Nivolumab in metastatic urothelial carcinoma after platinum therapy (CheckMate 275): a multicentre, single-arm, phase 2 trial. Lancet Oncol. 2017;18(3):312–22. doi: 10.1016/S1470-2045(17)30065-7 . 28131785

[7] Ferris RL, Blumenschein G Jr., Fayette J, Guigay J, Colevas AD, Licitra L, et al. Nivolumab for Recurrent Squamous-Cell Carcinoma of the Head and Neck. N Engl J Med. 2016;375(19):1856–67. doi: 10.1056/NEJMoa1602252 27718784 PMC5564292

[8] Kang YK, Boku N, Satoh T, Ryu MH, Chao Y, Kato K, et al. Nivolumab in patients with advanced gastric or gastro-oesophageal junction cancer refractory to, or intolerant of, at least two previous chemotherapy regimens (ONO-4538-12, ATTRACTION-2): a randomised, double-blind, placebo-controlled, phase 3 trial. Lancet. 2017;390(10111):2461–71. doi: 10.1016/S0140-6736(17)31827-5 . 28993052

[9] Scherpereel A, Mazieres J, Greillier L, Lantuejoul S, Do P, Bylicki O, et al. Nivolumab or nivolumab plus ipilimumab in patients with relapsed malignant pleural mesothelioma (IFCT-1501 MAPS2): a multicentre, open-label, randomised, non-comparative, phase 2 trial. Lancet Oncol. 2019;20(2):239–53. doi: 10.1016/S1470-2045(18)30765-4 . 30660609

[10] Ansell SM, Lesokhin AM, Borrello I, Halwani A, Scott EC, Gutierrez M, et al. PD-1 blockade with nivolumab in relapsed or refractory Hodgkin’s lymphoma. N Engl J Med. 2015;372(4):311–9. doi: 10.1056/NEJMoa1411087 25482239 PMC4348009

[11] El-Khoueiry AB, Sangro B, Yau T, Crocenzi TS, Kudo M, Hsu C, et al. Nivolumab in patients with advanced hepatocellular carcinoma (CheckMate 040): an open-label, non-comparative, phase 1/2 dose escalation and expansion trial. Lancet. 2017;389(10088):2492–502. doi: 10.1016/S0140-6736(17)31046-2 28434648 PMC7539326

[12] Rizzo A, Mollica V, Santoni M, Ricci AD, Rosellini M, Marchetti A, et al. Impact of Clinicopathological Features on Survival in Patients Treated with First-line Immune Checkpoint Inhibitors Plus Tyrosine Kinase Inhibitors for Renal Cell Carcinoma: A Meta-analysis of Randomized Clinical Trials. Eur Urol Focus. 2022;8(2):514–521. Epub 2021 Mar 11. doi: 10.1016/j.euf.2021.03.001 . 33714725

[13] Mollica V, Santoni M, Matrana MR, Basso U, De Giorgi U, Rizzo A, et al. Concomitant Proton Pump Inhibitors and Outcome of Patients Treated with Nivolumab Alone or Plus Ipilimumab for Advanced Renal Cell Carcinoma. Target Oncol. 2022;17(1):61–68. Epub 2021 Dec 11. doi: 10.1007/s11523-021-00861-y . 34894318

[14] Darnell EP, Mooradian MJ, Baruch EN, Yilmaz M, Reynolds KL. Immune-Related Adverse Events (irAEs): Diagnosis, Management, and Clinical Pearls. Curr Oncol Rep. 2020;22(4):39. doi: 10.1007/s11912-020-0897-9 . 32200442

[15] Sibaud V, Meyer N, Lamant L, Vigarios E, Mazieres J, Delord JP. Dermatologic complications of anti-PD-1/PD-L1 immune checkpoint antibodies. Curr Opin Oncol. 2016;28(4):254–63. doi: 10.1097/CCO.0000000000000290 . 27136138

[16] Williams SG, Mollaeian A, Katz JD, Gupta S. Immune checkpoint inhibitor-induced inflammatory arthritis: identification and management. Expert Rev Clin Immunol. 2020;16(8):771–85. doi: 10.1080/1744666X.2020.1804362 . 32772596

[17] Gonzalez-Rodriguez E, Rodriguez-Abreu D, Spanish Group for Cancer I-B. Immune Checkpoint Inhibitors: Review and Management of Endocrine Adverse Events. Oncologist. 2016;21(7):804–16. doi: 10.1634/theoncologist.2015-0509 27306911 PMC4943391

[18] Dougan M. Gastrointestinal and Hepatic Complications of Immunotherapy: Current Management and Future Perspectives. Curr Gastroenterol Rep. 2020;22(4):15. doi: 10.1007/s11894-020-0752-z . 32185493

[19] Sise ME, Seethapathy H, Reynolds KL. Diagnosis and Management of Immune Checkpoint Inhibitor-Associated Renal Toxicity: Illustrative Case and Review. Oncologist. 2019;24(6):735–42. doi: 10.1634/theoncologist.2018-0764 article. 30902916 PMC6656503

[20] Lyon AR, Yousaf N, Battisti NML, Moslehi J, Larkin J. Immune checkpoint inhibitors and cardiovascular toxicity. Lancet Oncol. 2018;19(9):e447–e58. doi: 10.1016/S1470-2045(18)30457-1 . 30191849

[21] Bukamur H, Katz H, Alsharedi M, Alkrekshi A, Shweihat YR, Munn NJ. Immune Checkpoint Inhibitor-Related Pulmonary Toxicity: Focus on Nivolumab. South Med J. 2020;113(11):600–5. doi: 10.14423/SMJ.0000000000001166 33140115 PMC7587235

[22] Cappelli LC, Gutierrez AK, Baer AN, Albayda J, Manno RL, Haque U, et al. Inflammatory arthritis and sicca syndrome induced by nivolumab and ipilimumab. Ann Rheum Dis. 2017;76(1):43–50. doi: 10.1136/annrheumdis-2016-209595 27307501 PMC5333990

[23] Kobayashi T, Iwama S, Yasuda Y, Okada N, Okuji T, Ito M, et al. Pituitary dysfunction induced by immune checkpoint inhibitors is associated with better overall survival in both malignant melanoma and non-small cell lung carcinoma: a prospective study. J Immunother Cancer. 2020;8(2). doi: 10.1136/jitc-2020-000779 32606047 PMC7328763

[24] Faje A, Reynolds K, Zubiri L, Lawrence D, Cohen JV, Sullivan RJ, et al. Hypophysitis secondary to nivolumab and pembrolizumab is a clinical entity distinct from ipilimumab-associated hypophysitis. Eur J Endocrinol. 2019;181(3):211–9. doi: 10.1530/EJE-19-0238 . 31176301

[25] Barroso-Sousa R, Ott PA, Hodi FS, Kaiser UB, Tolaney SM, Min L. Endocrine dysfunction induced by immune checkpoint inhibitors: Practical recommendations for diagnosis and clinical management. Cancer. 2018;124(6):1111–21. doi: 10.1002/cncr.31200 . 29313945

