# Supplementary material for: Utility of periodic medical questionnaires and examinations for immune-related adverse event screening: A prospective observational study
Source: PLoS One. 2022 Sep 29;17(9):e0274451. doi: 10.1371/journal.pone.0274451 (PMC9521803; doi:10.1371/journal.pone.0274451)
Supplement: Attachment — Submitted filename: Azuma et al_Reviewed by SD.pdf [file pone.0274451.s002.pdf]

Utility of periodic medical questionnaires and examinations for immune-related adverse event screening: a prospective observational study

Major Correction:

1. The statement required evidence: Please provide imaging results.

“Changes in ANA before and after immunotherapy were unknown in these three patients. Their report suggested that auto-antibodies were not useful for diagnosing inflammatory arthritis. On the other hand, imaging studies, such as ultrasonography or magnetic resonance imaging, are useful for diagnosing arthritis.”

Minor Corrections:

Several minor corrections have been detected and highlighted within the main manuscript PDF file. Please go through the Spacing related corrections.

Recommendation: Please use a tool that can potentially detect and help to correct grammatical errors.
