# Supplementary material for: Utility of periodic medical questionnaires and examinations for immune-related adverse event screening: A prospective observational study
Source: PLoS One. 2022 Sep 29;17(9):e0274451. doi: 10.1371/journal.pone.0274451 (PMC9521803; doi:10.1371/journal.pone.0274451)
Supplement: Attachment — Submitted filename: Reponse to Reviewers.docx [file pone.0274451.s003.docx]

**Reviewer #1**

 Despite ICI seem to have finally found their role in genitourinary malignancies, several questions remain unanswered. Among these, the lack of validated biomarkers of response represents an important issue since only a proportion of patients benefit from immunotherapy. Based on these premises, a greater understanding of the role of potential biomarkers including programmed death ligand 1 (PD-L1) expression, tumor mutational burden (TMB), microsatellite instability (MSI) status, gut microbiota and several others is fundamental. In addition, clinical trials on immunotherapy widely differed in terms of drugs, patients, designs, terms of study phases, and inconsistent clinical outcomes. In addition, ICIs have a specific set of toxicities, commonly called immune-related adverse events (irAEs), which are caused by the erroneous activation of the immune system against self-antigens. Multiple organ systems can be affected by irAEs, including the liver, and the incidence of hepatic irAEs varies depending on several factors, including the immunotherapeutic agent, tumor type, and disease setting.
Based on these premises, the study assesses a current, timely topic.
We recommend some changes:

- We believe this article is suitable for publication in the journal although major revisions are needed. The main strengths of this paper are that it addresses an interesting and very timely question and provides a clear answer, with some limitations. Certainly, the study is limited to an Asian population with a very small sample size, and authors should further express this point.

- A linguistic revision is needed.

***Thank you for your suggestion. We asked our in-house, native English-speaking editor to edit the manuscript.***

- Second, the study included a widely varied patient population from a single japanese institution and the total number of patients analyzed was relatively small. Thus, the authors should better highlight the limitations of the current paper.

***Thank you for your suggestion. We describe “The present study has a limitation. The study included a widely varied patient population from a single japanese institution and the total number of patients analyzed was relatively small” in Discussion.***

- The background of the changing scenario of medical treatment in genitoruinary malignnacies should be better discussed, and some recent papers regarding this topic should be included (PMID: 33714725; PMID: 34894318).

***Thank you for your suggestion. We cited two articles (PMID: 33714725; PMID: 34894318).***

**Reviewer #2**

Major Correction:

1. The statement required evidence: Please provide imaging results.

“Changes in ANA before and after immunotherapy were unknown in these three patients. Their　report suggested that auto-antibodies were not useful for diagnosing inflammatory arthritis. On　the other hand, imaging studies, such as ultrasonography or magnetic resonance imaging, are　useful for diagnosing arthritis.”

***Thank you for your remarks. We added an ultrasonography image in Figure 1 on page 16.***

Minor Corrections:

Several minor corrections have been detected and highlighted within the main manuscript PDF

***I could not find this PDF. We asked our in-house, native English-speaking editor to edit the manuscript.***

**Reviewer #3**

This is a fairly well written manuscript. As the authors have mentioned in the drawbacks, the practical adaptation and widespread implementation of questionnaires and examinations may not really be feasible. However, the authors present interesting correlations that can be useful for diagnosis and treatment making this manuscript and the overall study useful for the scientific community.

- It's not exactly clear from the manuscript the usage of the term "significant complaint". While it is described a few times across the manuscript, it is not clear how it is used in interpretation of the results or the discussion. Please clarify.

***Thank you for your suggestion. We described “The questionnaire was administered before the first ICI administration, on the day an ICI was administered, and one week later. The pre-ICI responses were used as the baseline against which the responses to the other two questionnaires were compared. A significant complaint was defined as a complaint not reported on the first questionnaire” in Materials and Methods.***

- Please change all numbers that are spelt out into numerical. Only a number at the beginning of the sentence needs to be spelt out. Others need to be numerical.

***Thank you for your suggestion. We change all numbers that are spelt out into numerical.***
